# One-Pot Hydrothermal Synthesis of Carbon Dots as Fluorescent Probes for the Determination of Mercuric and Hypochlorite Ions

**DOI:** 10.3390/nano11071831

**Published:** 2021-07-14

**Authors:** Hsin Lee, Yen-Chang Su, Hsiang-Hao Tang, Yu-Sheng Lee, Jan-Yee Lee, Cho-Chun Hu, Tai-Chia Chiu

**Affiliations:** 1 Department of Applied Science, National Taitung University, Taitung 95092, Taiwan; me.neolee@gmail.com (H.L.); 95695696a@gmail.com (Y.-C.S.); aaaa282911@gmail.com (H.-H.T.); ystw0206@gmail.com (Y.-S.L.); cchu@nttu.edu.tw (C.-C.H.); 2 Department of Environment Engineering, Kun Shan University, Tainan 710303, Taiwan; jylee@mail.ksu.edu.tw

**Keywords:** carbon dots, fluorescence quenching, hydrothermal, hypochlorite ion, mercuric ion, water samples

## Abstract

Nitrogen and sulfur codoped carbon dots (NSCDs) were synthesized via a one-pot hydrothermal method, and citric acid, ethylenediamine, and methyl blue were used as precursors. The obtained NSCDs were spherical with an average size of 1.86 nm. The fluorescence emission spectra of the NSCDs were excitation independent and emitted blue fluorescence at 440 nm with an excitation wavelength at 350 nm. The quantum yield of the NSCDs was calculated to be 68.0%. The NSCDs could be constructed as fluorescent probes for highly selective and sensitive sensing mercuric (Hg^2+^) and hypochlorite (ClO^−^) ions. As the addition of Hg^2+^ or ClO^−^ ions to the NSCDs, the fluorescence intensity was effectively quenched due to dynamic quenching. Under the optimal conditions, the linear response of the fluorescence intensity ranged from 0.7 μM to 15 μM with a detection limit of 0.54 μM and from 0.3 μM to 5.0 μM with a limit of detection of 0.29 μM for Hg^2+^ and ClO^−^ ions, respectively. Finally, the proposed method was successfully used for quantifying Hg^2+^ and ClO^−^ ions in spiked tap water samples.

## 1. Introduction

Carbon dots (CDs), a new kind of zero-dimensional fluorescent nanomaterial with sizes under 10 nm, have drawn tremendous attention from researchers since they were first discovered in 2004 [1]. Owing to their chemical inertness, stable photoluminescence, good water dispersibility, excellent biocompatibility, and ease of preparation and functionalization, CDs have wide applications in bioimaging [2,3], fluorescent sensors [4,5], photocatalysis [6], drug delivery [7], optoelectronic devices (light-emitting diodes and solar cells) [8], and therapy [9]. At present, various synthetic methods, such as arc discharge, laser ablation, electrochemical carbonization, ultrasonication, pyrolysis, microwave irradiation, and hydrothermal treatment, have been adopted for the fabrication of CDs. These methods can be simply classified into top-down and bottom-up strategies. Among these synthetic methods, the hydrothermal route has been the most common because it is simple, cost-effective, and convenient. However, the fluorescence quantum yield (QY) of the obtained CDs is relatively low (<5%). Doping with heteroatoms (e.g., B, N, S, and P) is considered to be an effective way to enhance the fluorescence properties of CDs [10]. Up to now, many efforts have been devoted to the synthesis of heteroatom-doped CDs from numerous precursors [11]. For example, Dong et al. [12] reported the preparation of nitrogen and sulfur codoped CDs (NSCDs) using citric acid and L-cysteine as precursors. The fluorescence quantum yield (QY) of the NSCDs was 80.0%. Wang et al. [13] employed citric acid and thiourea to fabricate NSCDs through hydrothermal synthesis. Results showed that the CDs had a bright blue emission with a QY of 73.1%. Zhang et al. [14] chose citric acid and *N*-aminoethylpiperazine as precursors to prepare nitrogen-doped CDs with a QY as high as 56.0%. Although the heteroatom-doped CDs had achieved high QYs, a facile hydrothermal treatment is still needed for the synthesis of fluorescent CDs from organic compounds with heteroatoms and specific functional groups for trace analysis.

Rapid detection of hazardous metal ions or anions in diverse environmental samples is very important for the protection of environments and human health. Mercury ions (Hg^2^^+^) are one of the most toxic pollutants owing to their high toxicity, lack of biodegradability, and easy bioaccumulation in the human body [15]. As a result, they pose a serious threat to human health and the environment. Hypochlorite ions (ClO^−^) are one of the most efficient oxidizing agents and have been extensively used in water treatment processes for destroying microorganisms [16]. However, the excess of ClO^−^ might react with organic compounds in aqueous solutions to produce undesirable trihalomethanes as byproducts. They have been reported to be harmful and carcinogenic [16]. Therefore, selective, sensitive, and efficient methods for Hg^2^^+^ or ClO^−^ ion detection in water samples are highly desirable. Up to now, fluorescent probes or fluorescence-based sensors have drawn much more attention due to their simplicity, low cost, high sensitivity, and short analysis time. Various fluorescent sensory materials are currently being developed and designed such as semiconductor quantum dots, metal nanoclusters and organic dye molecules [17,18]. However, these materials are restricted from use by their toxicity, low sensitivity, and high cost. Therefore, CDs could be an excellent candidate for the determination of Hg^2^^+^ or ClO^−^ ions [19,20,21,22,23,24]. For example, fluorescent CDs were synthesized from ascorbic acid and urea and used for the selective determination of Hg^2+^ and Cu^2+^ ions and bioimaging [19]. A fluorescence quenching sensor for Hg^2+^ based on CDs was designed by Yang et al. [20], which demonstrated improved selectivity based on the surface structural rational regulation. Wang et al. [21] reported multicenter-emitting CDs for the dynamic monitoring of wound-induced hypochlorous acid burst and oxidative stress in vivo. Nitrogen and phosphorus codoped CDs with long-wavelength emission have been synthesized and applied in sensing ClO^−^ and cell imaging [22]. Bruno et al. compared the interaction pathways with Hg^2+^ ions of the CDs synthesized through the bottom-up and top-down approaches and provided a convenient view for utilizing bottom-up CDs as sensors [25]. Indeed, an alternative fluorescent sensor is still required for the selective and sensitive detection of Hg^2^^+^ or ClO^−^ ions.

**Scheme 1:**
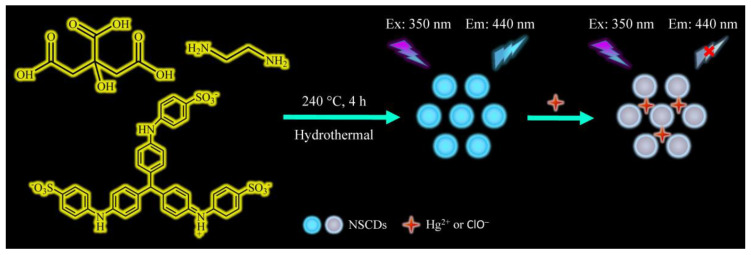
Preparation of sulfur and nitrogen codoped carbon dots (NSCDs) and their application in sensing mercury or hypochlorite ions through fluorescence quenching.

In this work, nitrogen and sulfur codoped carbon dots with strong blue fluorescence were synthesized through a hydrothermal treatment using citric acid, ethylenediamine, and methyl blue as carbon, nitrogen, and sulfur sources. NSCDs were used as a fluorescent sensor for Hg^2^^+^ and ClO^−^ ions, and the assay principle is shown in Scheme 1. The fluorescence emission of NSCDs was found to be independent of the excitation wavelength and insensitive to environmental conditions such as pH and ionic strength. The fluorescence intensity could be quenched by adding Hg^2^^+^ or ClO^−^ ions to the NSCDs. The selective and sensitive fluorescent probes were successfully developed for sensing Hg^2^^+^ or ClO^−^ ions in actual samples. Finally, UV–vis absorption spectroscopy and fluorescence decay measurements were used to analyze the fluorescence sensing mechanism for Hg^2^^+^ or ClO^−^ detection.

## 2. Materials and Methods

### 2.1. Chemicals and Reagents

Ascorbic acid, citric acid, disodium hydrogen phosphate, ethylenediamine, methyl blue, oxalic acid, phosphoric acid, quinine sulfate, sodium citrate, sodium dihydrogen phosphate, sodium hydroxide, trisodium phosphate, and the other metal salts used here were purchased from Sigma-Aldrich (St. Louis, MO, USA). Ultrapure water (≥18.2 MΩ cm) was used throughout all the experiments.

### 2.2. Characterization

The UV–vis absorption and fluorescence spectra were acquired by a U-2900 spectrophotometer (Hitachi, Tokyo, Japan) and an RF-6000 fluorescence spectrophotometer (Shimadzu, Kyoto, Japan). Fourier transform infrared (FT-IR) spectra were obtained on an FT-IR spectrometer (Perkin Elmer, Waltham, MA, USA) using the KBr pellet method. X-ray photoelectron spectroscopy (XPS) data were measured on a K-Alpha XPS system (Thermo Fisher Scientific, Waltham, MA, USA). Transmission electron microscopy (TEM) images were taken on a Philips CM-200 (Philips, Eindhoven, The Netherlands) with a working voltage of 200 kV.

### 2.3. Preparation of the NSCDs

The NSCDs were prepared through a one-pot hydrothermal approach. Briefly, citric acid (0.420 g) and methyl blue (0.003 g) were dissolved in ultrapure water (10 mL), and then ethylenediamine (536 μL) was added upon stirring for 10 min. The solution was subsequently transferred into a Teflon-lined stainless-steel autoclave and heated at 240 °C for 4 h. The resulting brown-color solution was purified by following the procedures in our previous research [26]. Finally, the purified NSCDs were stored at 4 °C for further characterization and applications.

### 2.4. Fluorescence Sensing of Hg^2+^ or ClO^−^

The NSCDs were applied for sensing Hg^2+^ or ClO^−^ ions. For a typical process, ultrapure water (600 μL), NSCD solution (200 μL), phosphate buffer (200 μL, 100 mM, pH 5.0), and various concentrations of Hg^2+^ or ClO^−^ (200 μL) were mixed well in 2 mL vials and reacted for 30 min. The fluorescence intensity of the NSCDs was recorded at 440 nm with an excitation wavelength of 350 nm. The working solutions of Hg^2+^ or ClO^−^ ions were freshly prepared by diluting stock solution (1 mM) with ultrapure water to the desired concentrations. The selectivity and interference measurements were operated at the same conditions mentioned above.

### 2.5. Determination of Hg^2+^ or ClO^−^ in Tap Water Samples

To evaluate the applicability of the NSCDs in practical applications for detecting of Hg^2+^ or ClO^−^ ions, three concentrations of Hg^2+^ (5.0 μM, 8.0 μM, and 10.0 μM) or ClO^−^ (0.5 μM, 2.0 μM, and 4.0 μM) were spiked in tap water samples. The water samples were centrifuged at 10,000 rpm for 10 min and then filtered through a 0.22 μm nitrocellulose filter to remove any suspended particles. The procedures for the quantitative analysis of Hg^2+^ or ClO^−^ ions were the same as Section 2.4.

## 3. Results and Discussion

### 3.1. Preparation and Characterization of the NSCDs

The NSCDs were synthesized using citric acid, ethylenediamine, and methyl blue as precursors through a one-pot hydrothermal approach. To obtain the NSCDs with excellent fluorescence properties, the effects of methyl blue content, temperature, and duration time for hydrothermal treatment were studied at several synthetic conditions. Table S1 shows the fluorescence QYs of the NSCDs under different synthetic conditions. It was found that hydrothermal treatment of 0.420 g of citric acid, 536 μL of ethylenediamine, and 0.003 g of methyl blue in 10 mL of ultrapure water at 240 °C for 4 h constituted optimal conditions. The calculated fluorescence QY of the NSCDs was raised up to 68.0% by using quinine sulfate (QY = 54%) as a standard [26], and they were used for further experiments as fluorescent probes. When in the presence of Hg^2+^ or ClO^−^ ions, the fluorescence intensity was quenched effectively and quickly. The morphology of the NSCDs was obtained by TEM that exhibited the NSCDs as spherical in shape and uniformly dispersed with an average diameter of 1.86 ± 0.04 nm (Figure S1).

**Figure 1:**
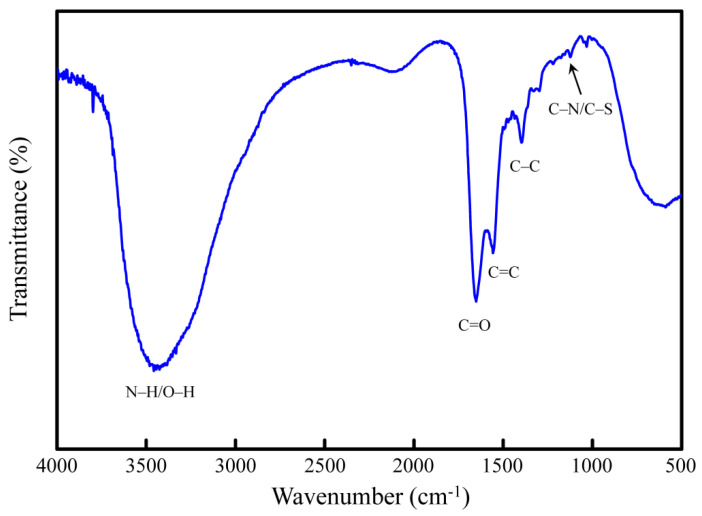
FT-IR spectrum of the NSCDs.

The surface functional groups of the NSCDs were characterized by FT-IR spectroscopy (Figure 1). The broad peak at 3000–3500 cm^–1^ was assigned to the stretching vibration absorption peak of the N–H and O–H bonds [27]. The peak at 1652 cm^–1^ was related to the stretching vibration peak of the C=O bond [28]. The bending vibration of the C=C appears at 1557 cm^–1^ [28]. The peak at 1397 cm^–1^ could be identified as the stretching vibration of the C–C bond [29]. The wake peak at 1124 cm^–1^ was attributed to the stretching vibration of the C–N and C–S bonds [30]. All these results confirmed that sulfur-, nitrogen-, and oxygen-containing functional groups were successfully introduced on the surface of the NSCDs.

**Figure 2:**
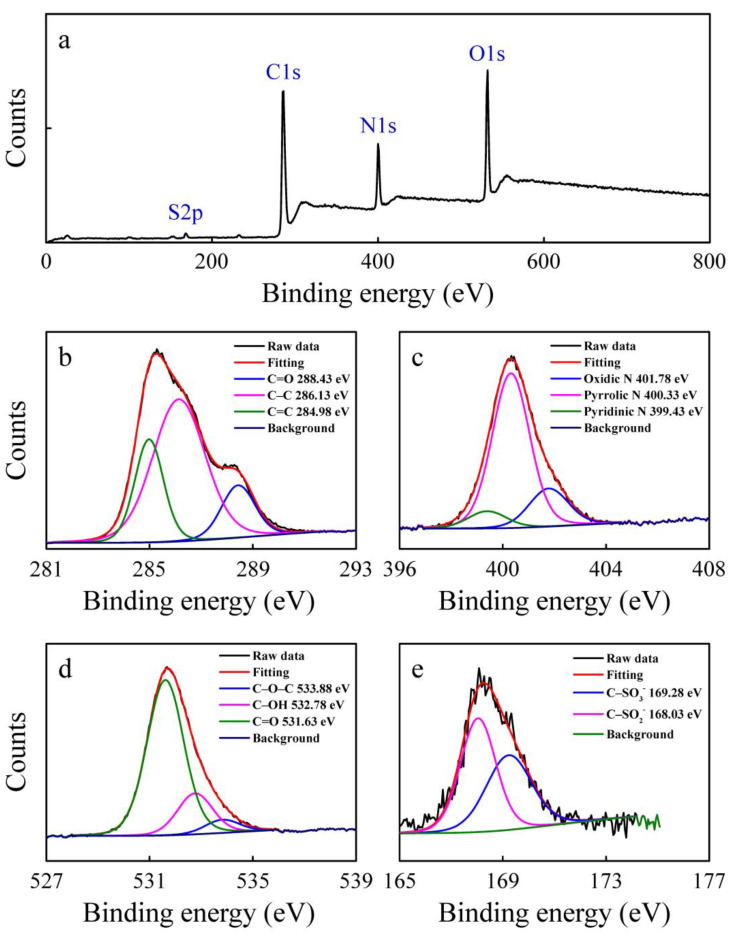
(**a**) XPS full survey spectra of the NSCDs. High-resolution XPS spectra of (**b**) C1s, (**c**) N1s, (**d**) O1s, and (**e**) S2p of the NSCDs.

The XPS measurements were further characterized as the elemental composition and chemical bonds of the NSCDs (Figure 2). The full-scan XPS spectrum (Figure 2a) of the NSCDs presented four peaks at 168.08 eV, 286.08 eV, 400.08 eV, and 532.08 eV, which corresponded to sulfur (S2p), carbon (C1s), nitrogen (N1s), and oxygen (O1s) elements [31], and their atomic contents were 1.09%, 65.99%, 14.17%, and 18.75%, respectively. The low content of sulfur atoms may be owing to the lower mole addition ratio of methyl blue (3.8 μmole) compared with citric acid (2.2 mmole) and ethylenediamine (7.9 mmole). These observations indicated that the NSCDs were composed of S, C, N, and O elements. Specifically, the high-resolution C1s spectrum (Figure 2b) was deconvoluted into three peaks at 284.98 eV, 286.13 eV, and 288.43 eV, which were attributed to C–C/C=C, C–O, and C=O groups, respectively [23]. The high-resolution N1s spectrum (Figure 2c) of the NSCDs showed three peaks detected at 399.43 eV, 400.33 eV, and 401.78 eV, which were attributed to pyridinic N, pyrrolic N, and graphitic N, respectively [27]. The peak of the high-resolution O1s spectrum (Figure 2d) could be fitted into three peaks at 531.63 eV, 532.78 eV, and 533.88 eV, which were assigned to O=C, O–H, and O=C–O groups, respectively [31]. As for the S2p spectrum (Figure 2e), the peaks centered at 168.03 eV and 169.28 eV were related to the –C–SO_x_– (x = 3 and 4) species [31].

### 3.2. Optical Properties of the NSCDs

**Figure 3:**
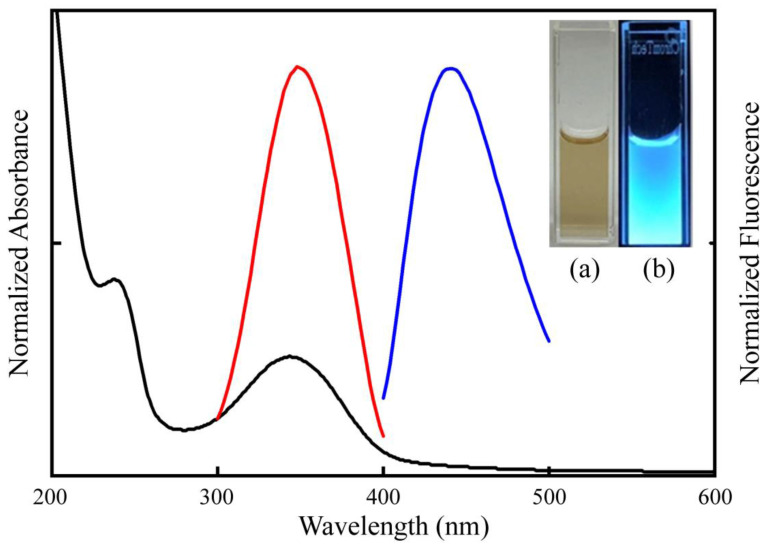
UV–vis absorption (black) and the maximum fluorescence excitation (red) and emission (blue) spectra of the NSCDs. Inset: Photos of the above suspension under (**a**) daylight and (**b**) UV irradiation (365 nm).

The UV–vis absorption and fluorescence spectra of the NSCDs were recorded to characterize their optical properties. Two obvious absorption peaks at 237 nm and 345 nm are shown in Figure 3, which could be attributed to the π–π* electronic transitions of the C=C bond and the n–π* electronic transitions of the C=O bond [32], respectively. The NSCDs showed a yellow color under daylight illumination and strong blue fluorescence excited by a UV lamp at 365 nm (inset of Figure 3). The excitation spectrum exhibited a characteristic peak at 350 nm, which corresponded to the UV–vis absorption spectrum, and the emission wavelength was observed at 440 nm. Moreover, the fluorescence spectra of the NSCDs were recorded under different excitation wavelengths from 320 nm to 380 nm. As a result, the emission wavelength almost remained at 440 nm with various excitation wavelengths (Figure S2). The excitation-independent fluorescence characteristic may be due to the narrow size distribution (Figure S1) and few surface defects on the NSCDs, which was similar to other reported CDs [12]. The NSCDs exhibited a fluorescence QY as high as 68.0%. The synergistic effect of the codoped sulfur and nitrogen atoms leads to the high-fluorescence QY [12].

### 3.3. Fluorescence Stability of the NSCDs

The effect of pH (2–12), ionic strength (0–1.0 M), UV irradiation time (0–60 min), and storage time (0–210 days) was employed to assess the fluorescence stability of the NSCDs. As shown in Figure S3, the NSCDs exhibited highly stable fluorescence intensity ranging from pH 4.0 to 10.0 (10 mM phosphate buffer) and at high ionic strength up to 1.0 M. The NSCDs also exhibited high photostability under a UV light irradiation for 60 min. Moreover, storage of the NSCDs for 7 months at 4 °C did not result in obvious aggregation, demonstrating the good stability of the NSCD solution. Therefore, these experimental results indicated potential applications of the NSCDs as effective fluorescent probes.

### 3.4. Fluorescence Quenching for Hg^2+^ or ClO^−^ Detection

**Figure 4:**
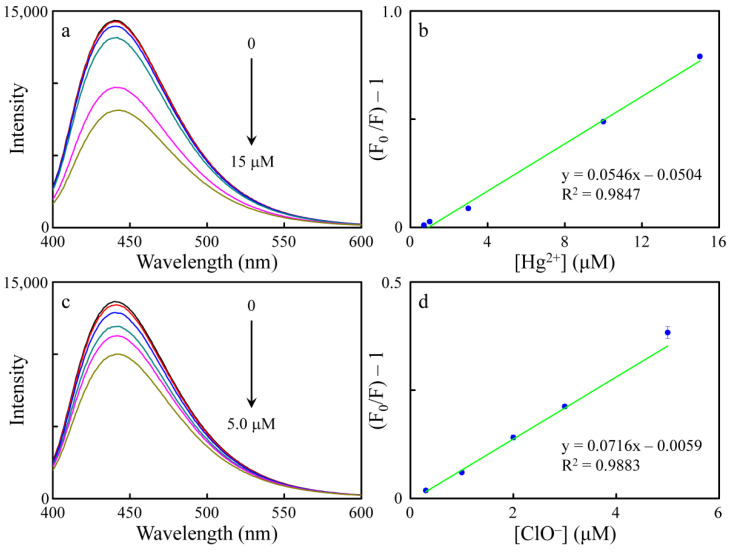
(**a**) Fluorescence emission spectra for the NSCDs in the presence of different concentrations of Hg^2+^. (**b**) Linear relationships between fluorescence intensity and concentrations of Hg^2+^ in the range of 0.7–15 μM. (**c**) Fluorescence emission spectra for the NSCDs in the presence of different concentrations of ClO^–^. (**d**) Linear relationships between fluorescence intensity and concentrations of ClO^–^ in the range of 0.3–5 μM.

To determine the concentration of Hg^2+^ or ClO^−^ ions in an aqueous solution, it was investigated by using the NSCDs as fluorescence probes. Figure 4 shows a gradually decreasing fluorescence intensity of the NSCDs at 440 nm with the increasing addition of Hg^2+^ or ClO^−^ ions. The fluorescence quenching efficiency of the NSCDs was evaluated through fitting the Stern−Volmer equation [33,34]:

F_0_/F = 1 + *K*_sv_ [Q] (1)

where F_0_ and F are the fluorescence intensity of the NSCDs at 440 nm in the absence and presence of the analytes (Hg^2+^ or ClO^−^ ions), respectively; *K*_sv_ is the Stern−Volmer constant; and Q is the concentration of the analytes. It was found (Figure 4b) that the proposed method showed a good linear relationship with Hg^2+^ concentration in the range of 0.7 μM to 15 μM, and a linear regression coefficient (R^2^ = 0.9847) and *K*_sv_ value of 5.46 × 10^4^ M^–1^ was obtained. Moreover, the calculated limit of detection (LOD) was 0.54 μM according to Equation 3σ/*m* (where σ is the standard deviation of the blank, *m* is the slope of the linear fit, and *n* = 10 independent measurements) [30]. Similarly, the fluorescence intensity was also obviously quenched by the addition of ClO^−^ to the NSCDs (Figure 4d). The fluorescence intensity ratio (F_0_/F) was proportional to the ClO^−^ concentration from 0.3 μM to 5 μM, and a linear regression coefficient (R^2^ = 0.9883) and *K*_sv_ value of 7.16 × 10^4^ M^–1^ was obtained. The LOD was calculated to be 0.29 μM (3σ/*m*). The proposed fluorescent probes were compared with other CD-based sensors for sensing Hg^2+^ or ClO^−^ ions, and the results are presented in Tables S2 and S3. As can be seen, the NSCDs had moderate sensitivity and a comparable linear range, thus indicating their potential applications for sensing Hg^2+^ or ClO^−^ ions.

**Figure 5:**
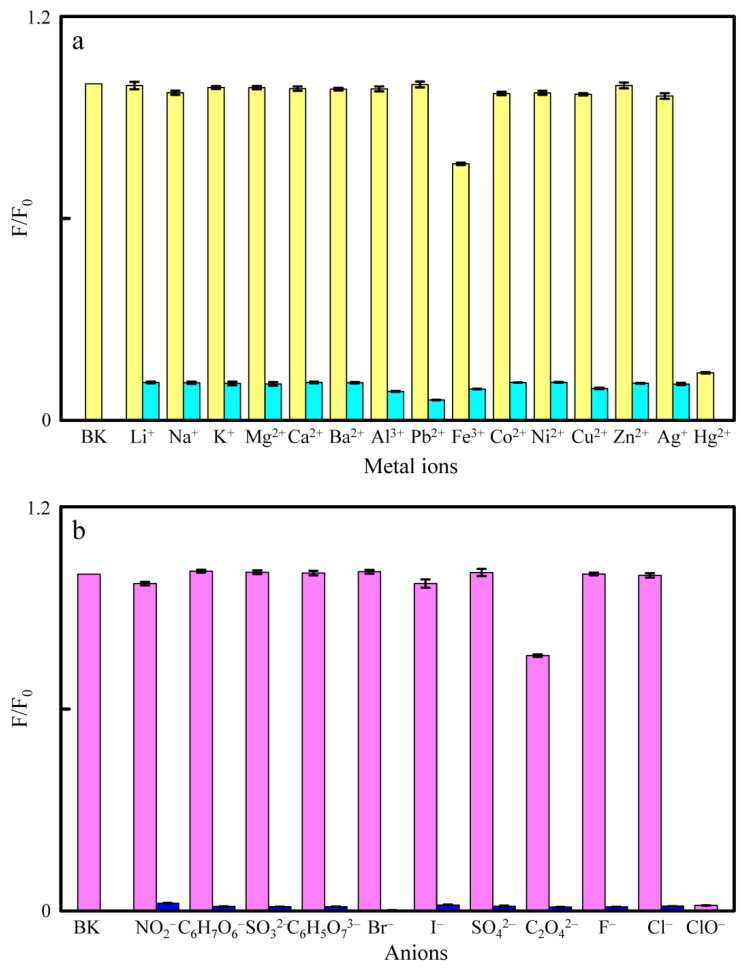
(**a**) The corresponding fluorescence intensity before (yellow) and after (cyan) the treatment of Hg^2+^. (**b**) The corresponding fluorescence intensity before (pink) and after (blue) the treatment of ClO^−^.

The selectivity experiments of the proposed method for Hg^2+^ or ClO^−^ ions detection were examined by recording the fluorescence response of the NSCDs toward some interfering ions (100 μM) with Hg^2+^ or ClO^−^ ions (100 μM). The fluorescence intensities of the NSCDs before and after the addition of the interfering ions were recorded. As shown in Figure 5a, the NSCDs showed a high affinity for the detection of Hg^2+^ under optimal conditions. Thus, Hg^2+^ was the only one that led to the obvious fluorescence quenching (85.9%) of the NSCDs, whereas the other metal ions caused slight or even no change. Similarly, among the possible interfering anions, they had almost no effect on the fluorescence intensity of the NSCDs, except with ClO^−^ (fluorescence intensity quenched 98.4%) (Figure 5b). These results proved that the NSCDs could be utilized as highly selective fluorescent probes for sensing Hg^2+^ or ClO^−^ ions.

The interference experiments revealed that the NSCDs were more selective toward Hg^2+^ and ClO^−^ compared to other possible coexisting ions (Figure 5). The concentrations of the possible interfering ions were tenfold higher than the concentrations of Hg^2+^ and ClO^−^ ions. It was clearly shown that the existence of other metal ions did not interfere with the detection of Hg^2+^. The interference experiment for other coexisting anions in the presence of ClO^−^ also obtained similar results. These observations indicated that the NSCDs could be utilized for the determination of Hg^2+^ or ClO^−^ ions selectively.

### 3.5. Sensing Mechanism

Generally, the fluorescence quenching of the NSCDs can be classified as a static or dynamic quenching mechanism [35]. These two mechanisms can be distinguished by the fluorescence lifetime and UV–vis absorption spectra of the NSCDs at various concentrations of the analytes. The fluorescence lifetime decay of the NSCDs in the absence and presence of Hg^2+^ or ClO^−^ ions were investigated (Figure S4). It was found that the measured average fluorescence lifetime of the NSCDs was 13.10 ns. After the addition of Hg^2+^ or ClO^−^, the fluorescence lifetime of the NSCDs was reduced to 9.16 ns (0.1 mM), 6.67 ns (1.0 mM), and 5.33 ns (10 mM) for NSCDs-Hg^2+^ and 11.13 ns (0.1 mM), 4.13 ns (1.0 mM), and 1.38 ns (10 mM) for NSCDs-ClO^−^, respectively. The phenomenon was similar to the previous report [36]. These results confirmed that the existence of Hg^2+^ or ClO^−^ ions alters the fluorescence lifetime of the NSCDs, indicating that the fluorescence quenching of the NSCDs by Hg^2+^ or ClO^−^ ions followed the dynamic quenching mechanism [35]. Figure S5 showed that the absorption threshold and peak position did not change after the addition of various concentrations of Hg^2+^ or ClO^−^ ions to the NSCDs in the wavelength range of 300 nm to 600 nm. The results indicated that Hg^2+^ or ClO^−^ did not interact with the NSCDs to form stable complexes. Therefore, non-fluorescence electron transfer from the excited state of the NSCDs to Hg^2+^ or ClO^−^ ions was the dominant fluorescence quenching mechanism [37].

### 3.6. Determination of Hg^2+^ or ClO^−^ in Water Samples

The applicability of the NSCDs was evaluated using Hg^2+^ (5.0 μM, 8.0 μM, and 10.0 μM) or ClO^−^ (0.5 μM, 2.0 μM, and 4.0 μM) spiked tap water samples by recording the fluorescence intensity change. The quenched fluorescence intensity was observed after the addition of Hg^2+^ or ClO^−^ ions. The satisfactory recoveries between 97.53% and 102.01% with RSDs below 4.27% for Hg^2+^ and between 93.67% and 116.49% with RSDs below 6.12% for ClO^−^ were obtained as shown in Table 1. The results clearly demonstrated that the NSCDs as fluorescence probes were applicable for the detection of Hg^2+^ and ClO^−^ ions in water samples.

## 4. Conclusions

In this study, NSCDs were successfully prepared through a facile one-pot hydrothermal approach wherein citric acid, ethylenediamine, and methyl blue were used as precursors. The blue fluorescence NSCDs with a high QY of 68.0% are well dispersed in water solutions. In addition, the NSCDs show highly sensitive and selective sensing Hg^2+^ and ClO^−^ ions through a fluorescence quenching process. The linear ranges and LODs are 0.7–15 μM and 0.54 μM for Hg^2+^ and 0.3–5 μM and 0.29 μM for ClO^−^, respectively. The results of UV–vis absorption spectra and fluorescence decay measurements indicate that the fluorescence quenching for sensing Hg^2+^ or ClO^−^ ions is related to the dynamic quenching mechanism. Therefore, the NSCDs can be used as fluorescent probes with high sensitivity and selectivity for the determination of Hg^2+^ or ClO^−^ ions in aqueous solutions.

## Figures and Tables

**Table 1:** Determination of Hg^2+^ and ClO^−^ ions in tap water samples (*n* = 3).

Analyte	Added (μM)	Found (μM)	Recovery (%)	RSD (%)
Hg^2+^	5.0	5.1	101.57	3.65
	8.0	7.8	97.53	4.11
	10.0	10.2	102.01	4.27
ClO^–^	0.5	0.5	116.49	6.12
	2.0	1.9	98.61	2.53
	4.0	3.8	93.67	2.80

## Data Availability

Data is contained within the article.

## Supplementary Materials

The following are available online at https://www.mdpi.com/article/10.3390/nano11071831/s1, Figure S1: TEM image and size distribution of the NSCDs, Figure S2: Fluorescence emission spectra of the NSCDs at different excitation wavelengths that change from 320 nm to 380 nm, Figure S3: Effect of various conditions on the fluorescence intensity of the NSCDs: (a) pH, (b) ionic strength, (c) UV light irradiation time, (d) storage time, Figure S4: Fluorescence decay curves of the NSCDs with (a) mercuric ions (0.1, 1.0, and 10 mM) and (b) hypochlorite ions (0.1, 1.0, and 10 mM), respectively, Figure S5: UV–vis absorption spectra of (a) Hg^2+^ and the NSCDs in the absence and presence of various concentrations of Hg^2+^, and (b) ClO^–^ and the NSCDs in the absence and presence of various concentrations of ClO^–^, Table S1: QYs of the NSCDs under different synthetic conditions, Table S2: Comparison of different fluorescent CDs applied for Hg^2+^ detection, Table S3: Comparison of different fluorescent CDs applied for ClO^–^ detection.
