# Supplementary material for: One-Pot Hydrothermal Synthesis of Carbon Dots as Fluorescent Probes for the Determination of Mercuric and Hypochlorite Ions
Source: Nanomaterials (Basel). 2021 Jul 14;11(7):1831. doi: 10.3390/nano11071831 (PMC8308378; doi:10.3390/nano11071831)
Supplement: Supplementary file 1 [file nanomaterials-11-01831-s001.zip › nanomaterials-1278070-supplementary.pdf]

Supplementary Materials

One-Pot Hydrothermal Synthesis of Carbon Dots as Fluorescent Probes for the Determination of Mercuric and Hypochlorite Ions

Hsin Lee [1], Yen-Chang Su [1], Hsiang-Hao Tang [1], Yu-Sheng Lee [1], Jan-Yee Lee [2], Cho-Chun Hu [1] and Tai-Chia Chiu [1,*]

[1] Department of Applied Science, National Taitung University, Taitung 95092, Taiwan; me.neolee@gmail.com (H.L.); 95695696a@gmail.com (Y.-C.S.); aaaa282911@gmail.com (H.-H.T.); ystw0206@gmail.com (Y.-S.L.); cchu@nttu.edu.tw (C.-C.H.)

[2] Department of Environment Engineering, Kun Shan University, Tainan 710303, Taiwan; jylee@mail.ksu.edu.tw

* Correspondence: tcchiu@nttu.edu.tw; Tel.: +886-89-517-990

Citation: Lee, H.; Su, Y.-C.; Tang, H.-H.; Lee, Y.-S.; Lee, J.-Y.; Hu, C.-C; Chiu, T.-C. One-Pot Hydrothermal Synthesis of Carbon Dots as Fluorescent Probes for the Determination of Mercuric and Hypochlorite Ions. *Nanomaterials* **2021**, *11*, 1831. https://doi.org/10.3390/nano11071831

Academic Editor: Antonios Kelarakis

Received: 12 June 2021
Accepted: 13 July 2021
Published: 14 July 2021

Publisher's Note: MDPI stays neutral with regard to jurisdictional claims in published maps and institutional affiliations.

Copyright: © 2021 by the authors. Submitted for possible open access publication under the terms and conditions of the Creative Commons Attribution (CC BY) license (https://creativecommons.org/licenses/by/4.0/).

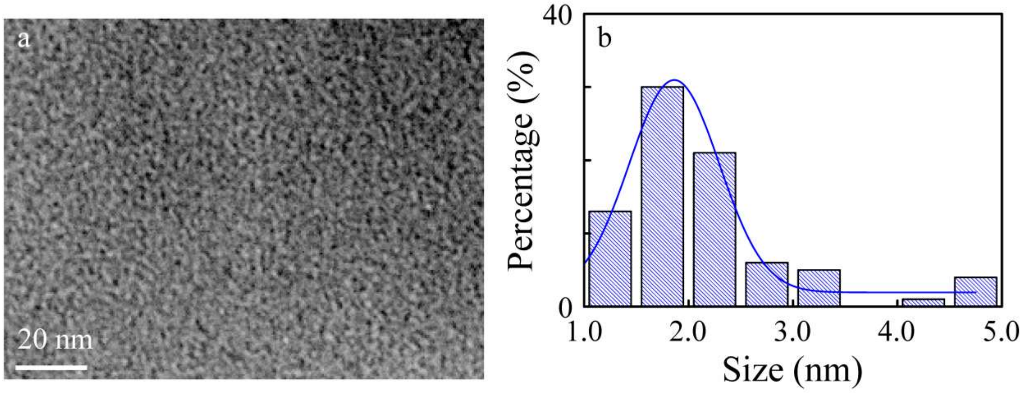

Figure S1. TEM image and size distribution of the NSCDs.

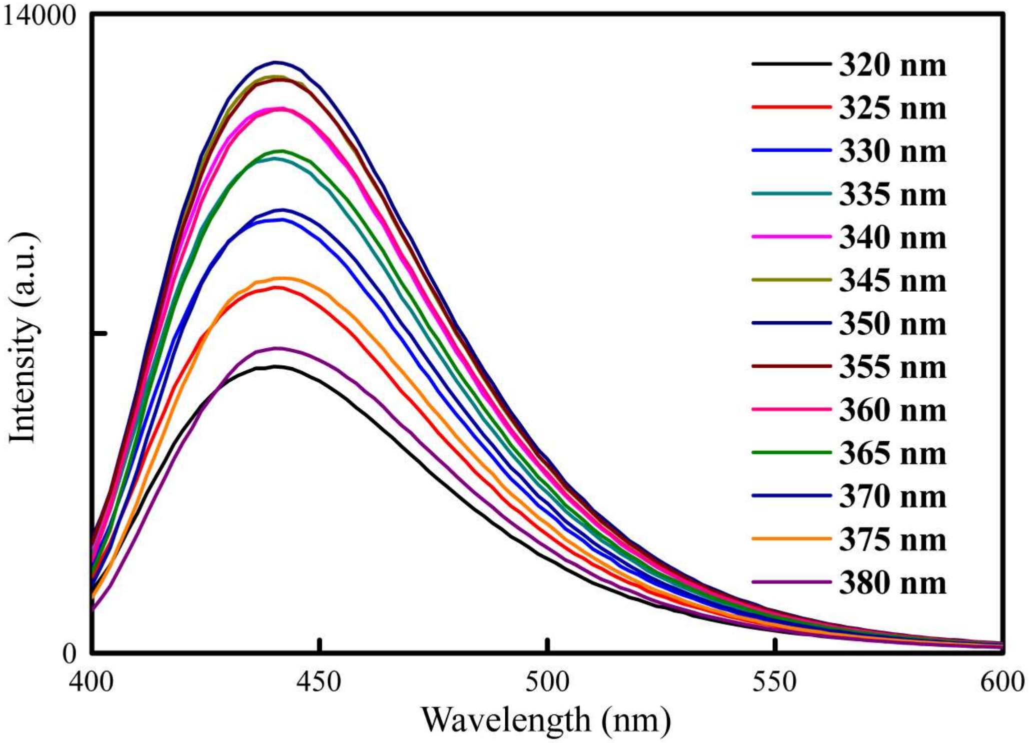

Figure S2. Fluorescence emission spectra of the NSCDs at different excitation wavelengths that change from 320 nm to 380 nm.

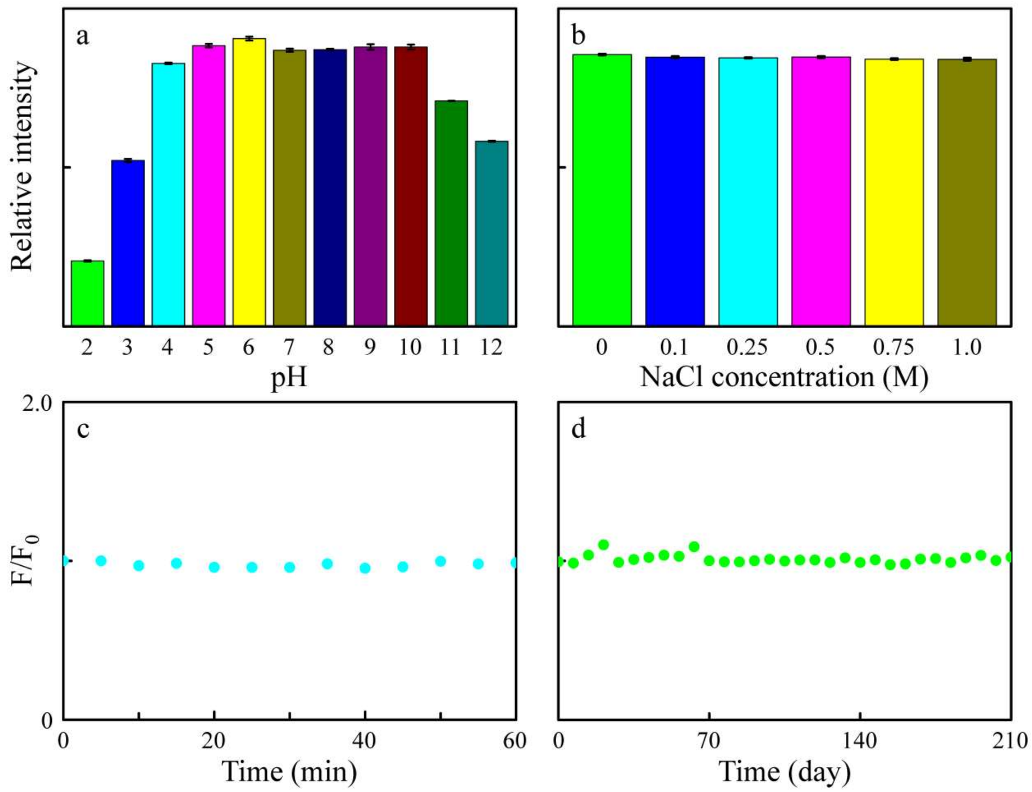

Figure S3. Effect of various conditions on the fluorescence intensity of the NSCDs: (**a**) pH, (**b**) ionic strength, (**c**) UV light irradiation time, (**d**) storage time.

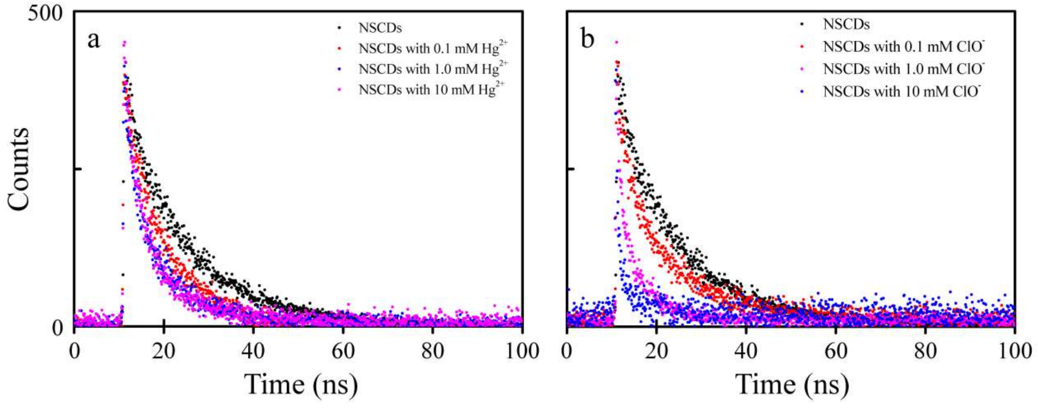

Figure S4. Fluorescence decay curves of the NSCDs with (**a**) mercuric ions (0.1, 1.0, and 10 mM) and (**b**) hypochlorite ions (0.1, 1.0, and 10 mM), respectively.

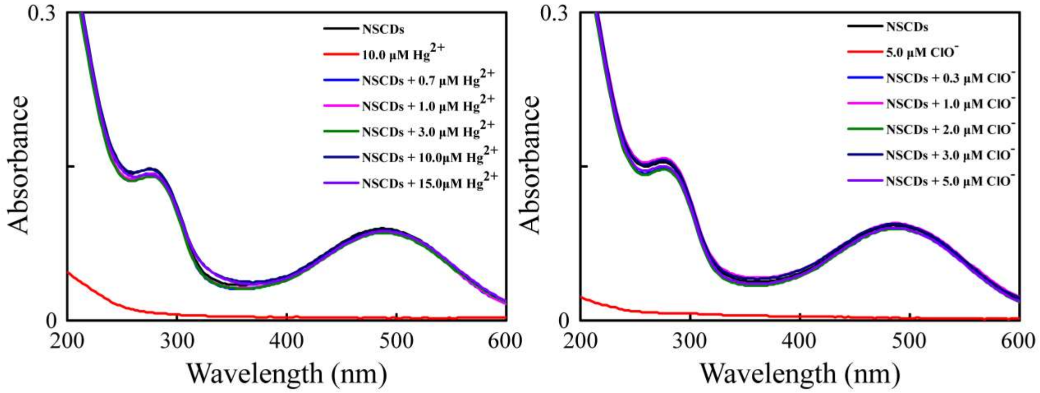

Figure S5. UV-Vis absorption spectra of (**a**) Hg^{2+} and the NSCDs in the absence and presence of various concentrations of Hg^{2+}, and (**b**) ClO^{-} and the NSCDs in the absence and presence of various concentrations of ClO^{-}.

Table S1. QYs of the NSCDs under different synthetic conditions.

Methyl blue (g)	Time (h)	Temperature (°C)	QY (%)
0	4	240	60
0.002	4	240	53
0.003	4	240	68
0.005	4	240	52
0.003	4	200	48
0.003	4	280	50
0.003	2	240	43
0.003	6	240	52

Table S2. Comparison of different fluorescent CDs applied for Hg^{2+} detection.

Probe	Precursors	QY (%)	Linear range (μM)	LOD (μM)	Ref.
N,S co-doped CDs	Ammonium citrate, L-cysteine	16.1	0.5–50	0.083	[1]
N-doped CDs	N-aminoethylpiperazine, citric acid	56.0	0–45	0.09	[2]
N,S co-doped CDs	Adipic acid, triammonium citrate	13.0	4–18	2.47	[3]
N,S co-doped CDs	Glycerol, cystine	3.5	1–75	0.5	[4]
N,S co-doped CDs	Citric acid, urea, L-cysteine	25.2	0–40	2	[5]
BU-CDs	Citric acid, urea	46.4	0.5–5	-	[6]
N,S co-doped CDs	Citric acid, ethylenediamine, methyl blue	68.0	0.7–15	0.54	This work

BU: bottom-up.

Table S3. Comparison of different fluorescent CDs applied for ClO^- detection.

Probe	Precursors	QY (%)	Linear range (μM)	LOD (μM)	Ref.
CDs	Sucrose, phosphoric acid	16.8	0.2–2.0	0.015	[7]
CDs	Sodium citrate, triethylenetetramine, rose	-	0–17	0.75	[8]
N, P co-doped CDs	Safranine T, phosphoric acid	15.6	0.74–5.93,	0.046	[9]
N, S co-doped CDs	*p*-phenylenediamine, cysteamine	-	0.18–4.22	0.021	[10]
N, S co-doped CDs	Glutathione, urea	8.6	60–150	3.4	[11]
N, S co-doped CDs	Citric acid, ethylenediamine, methyl blue	68.0	0.3–5	0.29	This work

References:

1. Wu, H.; Tong, C. Nitrogen- and sulfur-codoped carbon dots for highly selective and sensitive fluorescent detection of Hg^{2+} ions and sulfide in environmental water samples. *J. Agric. Food Chem.* **2019**, *67*, 2794–2800.
2. Zhang, H.; You, J.; Wang, J.; Dong, X.; Guan, R.; Cao, D. Highly luminescent carbon dots as temperature sensors and "off-on" sensing of Hg^{2+} and biothiols. *Dyes Pigments* **2020**, *173*, 107950.
3. Yan, F.; Kong, D.; Luo, Y.; Ye, Q.; He, J.; Guo, X.; Chen, L. Carbon dots serve as an effective probe for the quantitative determination and for intracellular imaging of mercury(II). *Microchim. Acta* **2016**, *183*, 1611–1618.
4. Xu, Y.; Li, H.; Wang, B.; Liu, H.; Zhao, L.; Zhou, T.; Liu, M.; Huang, N.; Li, Y.; Ding, L., et al. Microwave-assisted synthesis of carbon dots for "turn-on" fluorometric determination of Hg(II) via aggregation-induced emission. *Microchim. Acta* **2018**, *185*, 252.

5. Li, L.; Yu, B.; You, T. Nitrogen and sulfur co-doped carbon dots for highly selective and sensitive detection of Hg(II) ions. *Biosens. Bioelectron.* **2015**, *74*, 263–269.
6. Bruno, F.; Sciortino, A.; Buscarino, G.; Soriano, M.L.; Ríos, Á.; Cannas, M.; Gelardi, F.; Messina, F.; Agnello, S. A Comparative Study of Top-Down and Bottom-Up Carbon Nanodots and Their Interaction with Mercury Ions. *Nanomaterials* **2021**, *11*, 1265.
7. Huang, Z.; Lin, F.; Hu, M.; Li, C.; Xu, T.; Chen, C.; Guo, X. Carbon dots with tunable emission, controllable size and their application for sensing hypochlorous acid. *J. Lumin.* **2014**, *151*, 100–105.
8. Zhang, C.; Liu, M.; Li, T.; Liu, S.; Chen, Q.; Zhang, J.; Zhang, K. One-pot hydrothermal synthesis of dual-emission fluorescent carbon dots for hypochlorous acid detection. *Dyes Pigments* **2020**, *180*, 108507.
9. Jiao, Y.; Meng, Y.; Lu, W.; Gao, Y.; Liu, Y.; Gong, X.; Liu, Y.; Shuang, S.; Dong, C. Design of long-wavelength emission carbon dots for hypochlorous detection and cellular imaging. *Talanta* **2020**, *219*, 121170.
10. Zhang, Z.; Pei, K.; Yang, Q.; Dong, J.; Yan, Z.; Chen, J. A nanosensor made of sulfur–nitrogen co-doped carbon dots for "off–on" sensing of hypochlorous acid and Zn(II) and its bioimaging properties. *New J. Chem.* **2018**, *42*, 15895–15904.
11. Wang, R.; Wang, R.; Ju, D.; Lu, W.; Jiang, C.; Shan, X.; Chen, Q.; Sun, G. "ON–OFF–ON" fluorescent probes based on nitrogen-doped carbon dots for hypochlorite and bisulfite detection in living cells. *Analyst* **2018**, *143*, 5834–5840.
